# Central role of mTORC1 downstream of YAP/TAZ in hepatoblastoma development

**DOI:** 10.18632/oncotarget.20622

**Published:** 2017-09-01

**Authors:** Pin Liu, Diego F. Calvisi, Andras Kiss, Antonio Cigliano, Zsuzsa Schaff, Li Che, Silvia Ribback, Frank Dombrowski, Dongchi Zhao, Xin Chen

**Affiliations:** ^1^ Department of Pediatrics, Zhongnan Hospital of Wuhan University, Wuhan, Hubei, China; ^2^ Department of Bioengineering and Therapeutic Sciences, Liver Center, University of California San Francisco, San Francisco, CA, USA; ^3^ Institute of Pathology, University of Greifswald, Greifswald, Germany; ^4^ Second Department of Pathology, Semmelweis University, Budapest, Hungary

**Keywords:** YAP/TAZ, mTORC1, SLC38A1, hepatoblastoma

## Abstract

Hepatoblastoma (HB) is the most common type of liver malignancy in children. Recent studies suggest that activation of Yes-associated protein (YAP) is a major molecular event in HB development, as activated YAP synergizes with mutant β-catenin to promote HB formation in mice (YAP/β-catenin). However, how YAP regulates HB development remains poorly defined. Similarly, de-regulation of mammalian target of rapamycin complex 1 (mTORC1) signaling has been implicated in multiple tumor types, but its functional role in HB development is scarcely understood. In the present study, we found that mTORC1 is activated in human HB cells and YAP/β-catenin-induced mouse HB tumor tissues. mTOR inhibitor MLN0128 significantly inhibits human HB cell growth *in vitro*. Furthermore, ablation of *Raptor*, the unique subunit of mTORC1, strongly delayed YAP/β-catenin-induced HB development in mice. At the molecular level, we found that expression of the amino acid transporter SLC38A1 is induced in mouse HB tissues, and amino acid deprivation leads to mTORC1 suppression in HB cell lines. Silencing of YAP and/or its paralog, transcriptional co-activator with PDZ binding motif (TAZ), decreased SLC38A1 expression as well as mTORC1 activation in HB cells. Furthermore, a frequent and concomitant upregulation of mTORC1 and SLC38A1 was detected in a collection of human HB specimens. Altogether, our study demonstrates the key role of mTORC1 in HB development. YAP and TAZ promote HB development *via* inducing SLC38A1 expression, whose upregulation leads to mTORC1 activation. Targeting mTOR pathway or amino acid transporters may represent novel therapeutic strategies for the treatment of human HB.

## INTRODUCTION

Hepatoblastoma (HB) is the most common liver malignancy in children. HB usually occurs in the first 3 years of life. The incidence is slowly but steadily increasing at a rate of 1.2-1.5 cases/million/year, partly due to prematurity and very low birth weight [1, 2]. The development of adjuvant, neo-adjuvant chemotherapy as well as the new surgical resection methods significantly improved the patients’ survival rate in the last decades [3]. Despite these significant achievements in HB treatment, the prognosis of patients with metastasis and those who are at a stage of pretreatment extent of disease (PRETEXT) IV remains unfavorable [4]. Thus, novel targeted therapy strategies against HB are highly needed. To achieve this goal, a better characterization of the molecular genetics and signaling pathways underlying HB pathogenesis is imperative.

Using exome sequencing approaches, recent studies have identified multiple genetic modifications in human HB samples, including gain-of-function mutations of *CTNNB1* and *CAPRIN2*, and loss-of-function mutations of *Axin1* and *SPOP* [5]. Among them, mutations of *CTNNB1* and *CAPRIN2* genes lead to the activation of the canonical Wnt pathway, a major driver genetic event promoting HB development [6, 7]. Point mutations as well as in-frame deletions of the exon 3 in *CTNNB1* gene have been detected in ∼80% of human HB patients. These mutations lead to stabilization, nuclear localization, and activation of β-catenin transcriptional activity, with consequent induction of a large number of target genes involved in proliferation, survival, migration, and invasion [8, 9]. It is important to underline that while β-catenin cascade is essential for HB development [10], over-expression of either full length, N-terminal deleted mutation (ΔN90), or point mutation (S45Y) forms of β-catenin alone is not sufficient to promote HB formation in mice. This evidence suggests that activation of additional signaling pathways is required, and these pathways may synergize with activated Wnt/β-catenin cascade to promote HB development [11]. In a recent study from our group, we found that Yes-associated protein (YAP), the transcriptional co-activator downstream of the Hippo tumor suppressor pathway, is activated in most human HB samples, with coordinated activation of YAP and β-catenin being detected in ∼80% of HB [11]. Importantly, co-expression of activated YAP and β-catenin in the mouse liver *via* hydrodynamic transfection led to HB formation in mice [11]. Our study, therefore, establishes YAP as a second signal that synergizes with β-catenin to promote HB development. However, the molecular mechanisms whereby YAP promotes HB formation remain unknown.

The mammalian target of rapamycin (mTOR) pathway is a central regulator of tissue growth and homeostasis [12]. It is comprised of two multiple protein complexes: mTOR complex 1 (mTORC1) and 2 (mTORC2), with regulatory-associated protein of mammalian target of rapamycin (Raptor) and rapamycin-insensitive companion of mTOR (Rictor) as unique protein components for mTORC1 and mTORC2, respectively [12]. De-regulation of the mTOR pathway is frequently found in human cancers [13], including hepatocellular carcinoma (HCC), the most common form of primary liver cancer [14, 15]. Temsirolimus, a first generation mTOR inhibitor, is approved by the Food and Drug Administration (FDA) for the treatment of advanced stage renal cell carcinoma [16]. Similarly, targeting the mTOR signaling has been considered a promising strategy for the treatment of HCC [17]. However, studies on the functional contribution of the mTOR pathway to HB development are lacking.

In the present study, we found that mTORC1 is activated in human HB cell lines as well as YAP/β-catenin-induced mouse HB tissues. A key role of mTORC1 in HB development was substantiated by subsequent functional studies. Mechanistically, we found that YAP induces the expression of the amino acid transporter SLC38A1, leading to the activation of mTORC1. Therefore, our study strongly suggests that mTORC1 is a major signaling event downstream of activated YAP along HB development. The results obtained also support the further testing of mTOR inhibitors for the treatment of human HB.

## RESULTS

### Activation of mTORC1 in human and mouse hepatoblastoma cells

**Figure 1:**
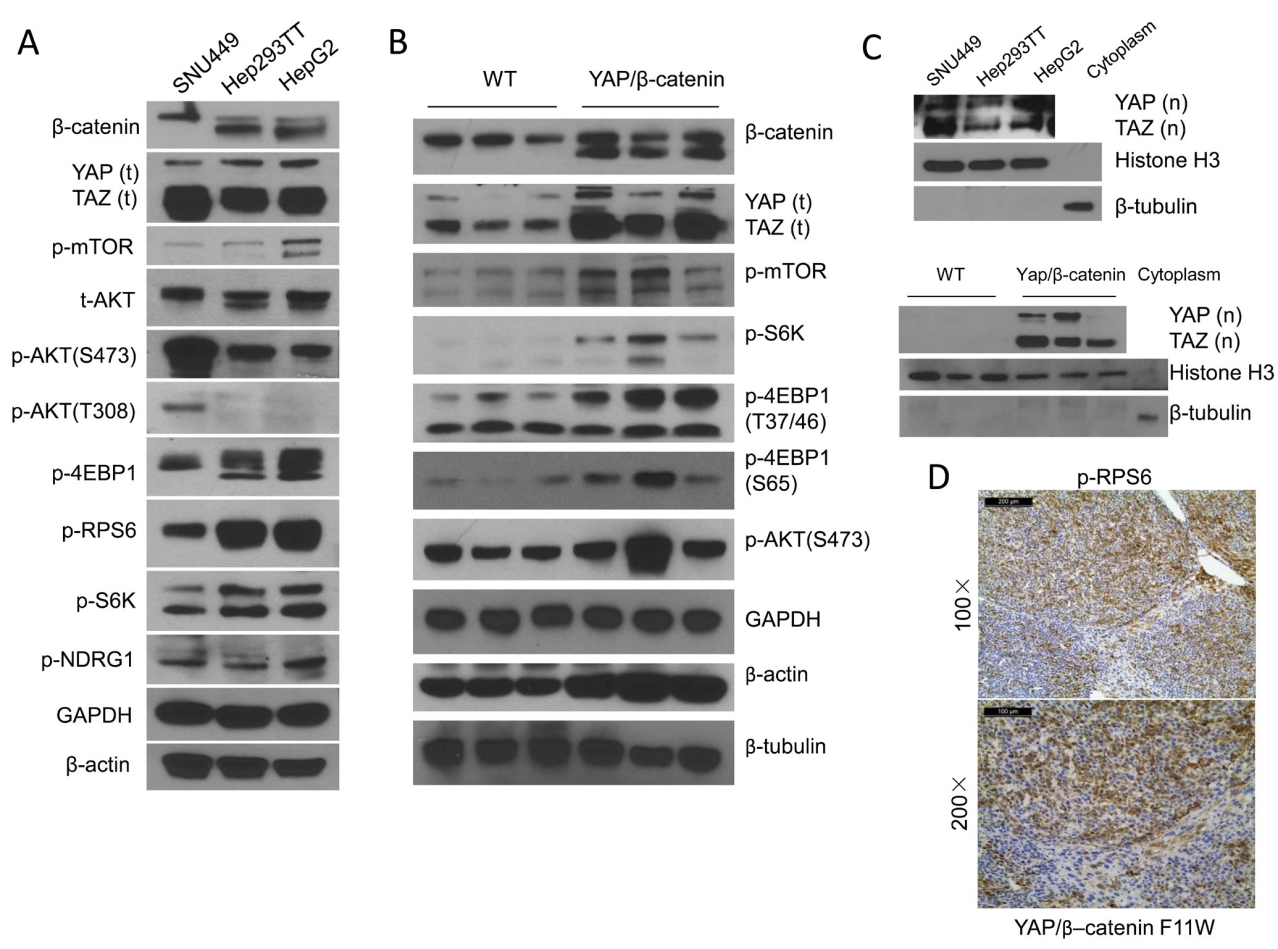
mTORC1 is activated in human hepatoblastoma (HB) cells and YAP/β-catenin driven HB lesions **A.** Representative Western blotting of YAP, TAZ, β-catenin as well as proteins from the AKT/mTOR pathway in Hep293TT and HepG2 HB cells, and SNU449 HCC cells. **B.** Representative Western blotting from wild-type (WT) and YAP/β-catenin-induced mouse HB samples. **C.** Nuclear expression of YAP [YAP (n)] and TAZ [TAZ (n)] in the human HB cell lines (upper level) and mouse HB tissues (lower level). Histone H3 was used as loading control for nuclear extraction; and β-tubulin as loading control for cytoplasmic extraction. **D.** Immunohistochemistry showing strong upregulation of phosphorylated (p-)/activated RPS6 (a surrogate marker of mTORC1 activation) in YAP/β-catenin-induced mouse HB samples. Magnifications: upper level: 100×, scale bar = 200μm; Lower level: 200×, scale bar = 100μm.

As a first step to study the functional crosstalk between YAP and mTORC1 in HB, we analyzed the protein levels of β-catenin, YAP, TAZ (a paralog of YAP), and members of the mTOR pathway using two human HB cell lines, namely Hep293TT and HepG2 cells [18, 19]. The SNU449 human HCC cell line that harbors a mutation in the Pten tumor suppressor gene (thus displaying a constitutively high mTOR activity) was used as positive control. Both Hep293TT and HepG2 HB cell lines have N-terminal truncated forms of β-catenin, which could be readily detected as a lower band by Western blotting (Figure 1A). Total and nuclear YAP and TAZ were found to be expressed in the three cell lines, supporting the activation of the two protooncogenes in these cells (Figure 1A and 1C). Activation of mTORC1 in Hep293TT and HepG2 cells was evidenced by the elevated levels of phosphorylated/activated (p)-mTOR, phosphorylated/inactivated (p)-4EBP1, phosphorylated/activated (p)-S6K and phosphorylated/activated (p)-RPS6 (Figure 1A). Furthermore, levels of mTORC2 targets, including phosphorylated (p)-NDRG1 and phosphorylated/activated (p)-AKT (S473) were elevated, thus indicating the activation of mTORC2 in Hep293TT and HepG2 cells. In contrast, levels of PDK1 substrate phosphorylated/activated (p)-AKT (T308) were very low/undetectable by Western blotting in Hep293TT and HepG2 cell lines, whereas they were induced in SNU449 HCC cells (Figure 1A). Next, we analyzed the expression of these pathways in YAP/β-catenin-induced mouse HB tumor tissues (Figure 1B-1D). Consistently, truncated β-catenin and nuclear YAP and TAZ could be found in mouse HB tissues. In addition, levels of p-mTOR, p-S6K, p-RPS6 and p-4EBP1 were all higher in HB tumor tissues than normal liver tissues, supporting the activation of mTORC1 in YAP/β-catenin-induced HB tumors.

In summary, these findings indicate the activation of mTORC1 in addition to activated Wnt/β-catenin and YAP/TAZ in human and mouse HB cells.

### The mTOR inhibitor MLN0128 suppresses HB cell growth *in vitro*

**Figure 2:**
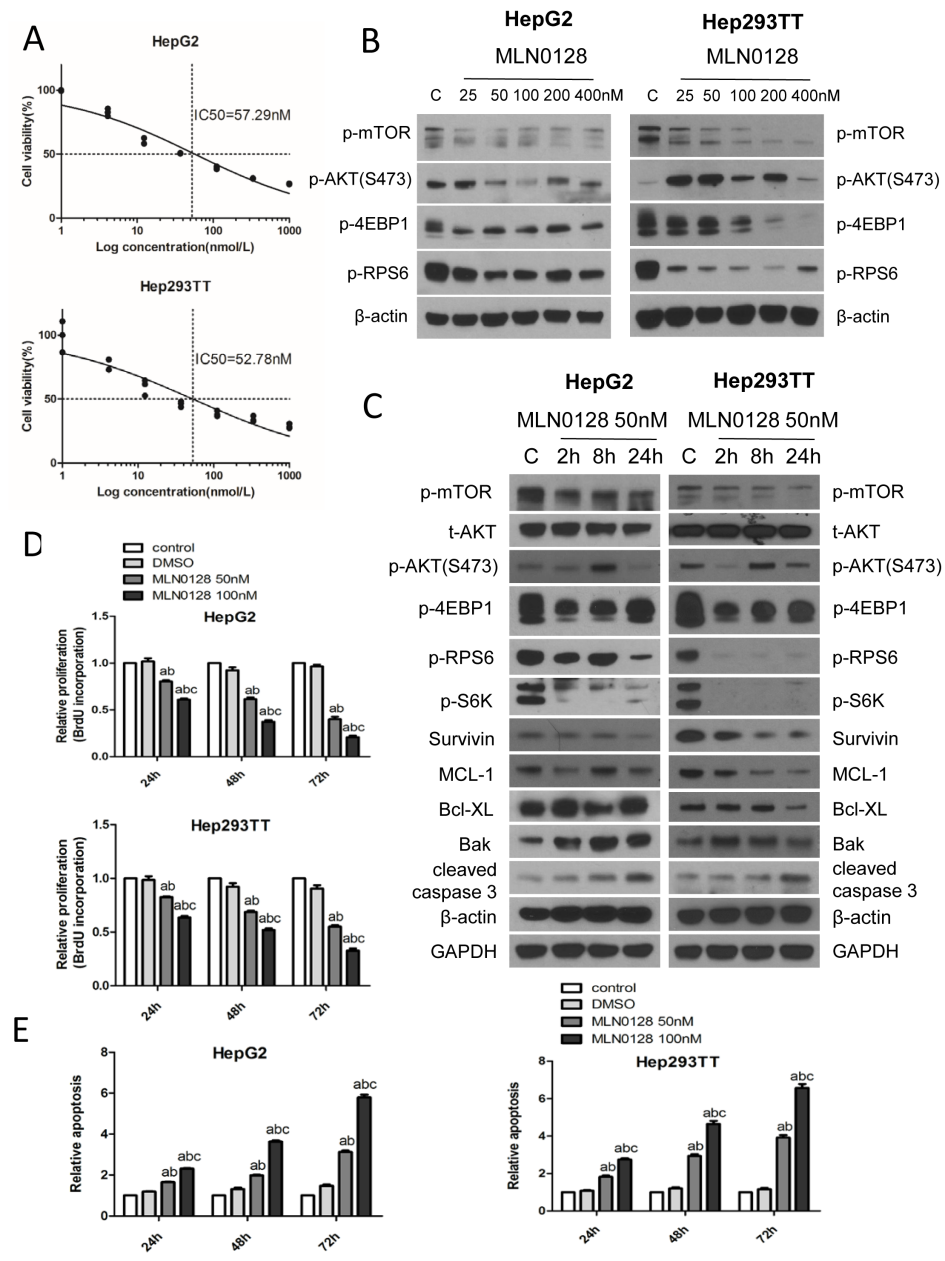
MLN0128 inhibits HB cell growth *via* targeting the mTOR pathway **A.** IC_50_ of MLN0128 for HepG2 and Hep293TT HB cells. **B.** Western blotting showing the downregulation of mTORC1 upon various concentrations of MLN0128 treatment in HepG2 and Hep293TT cell lines. **C.** Western blotting of mTOR- and apoptosis pathway-related proteins in HepG2 and Hep293TT cells treated with MLN0128 at its IC_50_ concentration. Graph showing **D.** BrdU incorporation based proliferation analysis and **E.** relative apoptosis after various concentrations of MLN0128 treatment at different time courses (24, 48 and 72 hours) in HepG2 and Hep293TT cell lines. Data are presented as mean ± SEM. *** *P* < 0.001; **A.** *vs* control (untreated cells); **B.** *vs* DMSO; and **C.** *vs* MLN0128 50nM.

Next, we determined whether HB cells are sensitive to mTOR inhibitors. For this purpose, we chose MLN0128, a second generation mTOR inhibitor [20]. Unlike allosteric mTOR inhibitors (such as Rapamaycin), which partially inhibit mTORC1, MLN0128 has been shown to complete suppress mTORC1 activity as indicated by the strong inhibition of p-4EBP1 [20]. Importantly, both Hep293TT and HepG2 cells were found to be highly sensitive to MLN0128, with an IC_50_ around 50nM (Figure 2A). At the molecular level, administration of MLN0128 led to an increased inhibition of mTORC1 targets, including p-mTOR, p-RPS6 and p-4EBP1, in a dose-dependent manner (Figure 2B). In the time course study, sustained inhibition of p-mTOR, p-S6K, p-RPS6 and p-4EBP1 was detected when Hep293TT and HepG2 cells were treated with MLN0128 at 50nM (Figure 2C). Furthermore, MLN0128 dramatically decreased cell proliferation and increased cell apoptosis in a dose-dependent manner (Figure 2D and 2E). Consistently, pro-survival proteins, including Survivin and MCL-1, were found to be decreased, whereas the pro-apoptotic protein Bak was upregulated in MLN0128 treated HB cell lines. Consistent with increased apoptosis, cleaved caspase 3 levels were highest in Hep293TT and HepG2 cells treated with MLN0128 (Figure 2C).

Altogether, the present data indicate that mTOR inhibitor MLN0128 suppresses HB cell growth *in vitro*.

### Ablation of *Raptor* strongly inhibits YAP/β-catenin-induced HB tumorigenesis in mice

**Figure 3:**
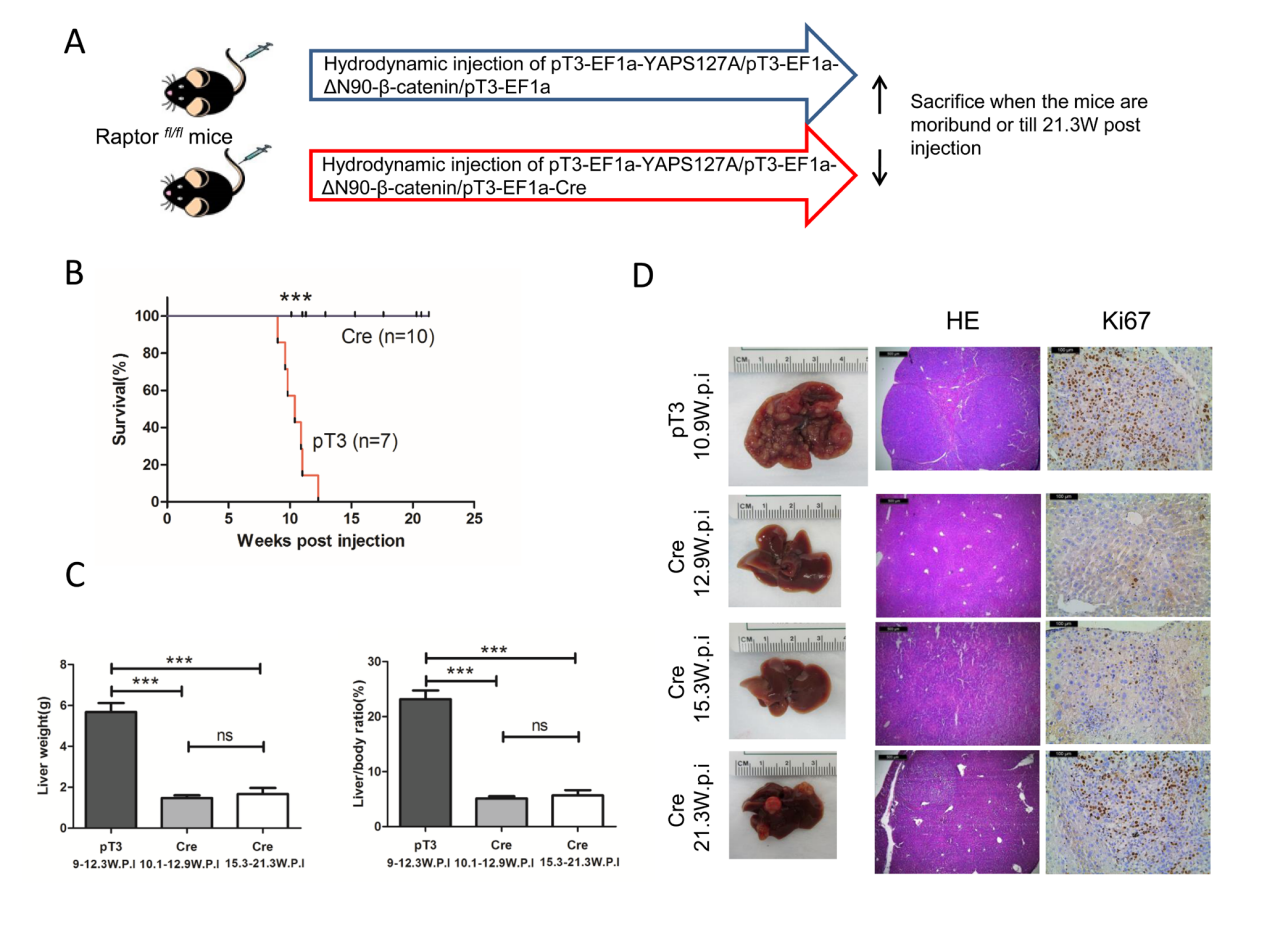
Knocking out Raptor strongly delays YAP/β-catenin-induced HB development in mice **A.** Study design. **B.** Survival curve. **C.** Liver weight and liver/body weight of YAP/β-catenin/pT3EF1α (pT3) and YAP/β-catenin/pT3EF1α-Cre (Cre) injected mice. Data are presented as mean ± SEM. *** *P* < 0.001; ns, not significant. **D.** Gross images of livers, HE and Ki67 staining in pT3 and Cre mouse livers. Magnifications: 40× (HE), scale bar = 500μm; 200× (Ki67), scale bar = 100μm.

Since Raptor is the unique and functional component of mTORC1, we performed Cre/LoxP mediated *Raptor* knockout by hydrodynamic tranfection in *Raptor*^*fl/fl*^ mice. The study design is similar to what we have published previously [21]. In brief, either pT3-EF1a (pT3, empty vector control) or pT3-EF1a-Cre (Cre) plasmid was co-injected with YAPS127A and ΔN90-β-catenin plasmids into *Raptor*^*fl/fl*^ mice (Figure 3A). Mice were monitored and sacrificed when they were moribund or 21.3 weeks post injection. All the pT3 treated mice developed a lethal burden of liver tumors by 12.3 weeks post injection and were euthanized. In striking contrast, all Cre injected mice remained healthy until 21.3 weeks post injection (Figure 3B and Table 1). Upon dissection, within 10 to 12 weeks post injection, all pT3 injected mice showed massive liver tumors with high liver weight as well as liver/body ratio (Figure 3C and 3D). Histologically, tumor cells resembled fetal or crowded fetal subtype of human HB, characterized by small cell size as well as small round or oval nuclei [11]. Tumor cells were highly proliferative, as indicated by Ki67 staining. At this time point, all Cre injected mice showed the absence of tumor lesions on the liver surface, with normal liver weight and liver/body ratio (Figure 3C and 3D). Histological examination revealed that the liver of Cre injected mice was completely normal with no microscopic lesions (Figure 3D). At 15 to 21 weeks post injection, small tumor nodules could be found in Cre injected mouse livers (Figure 3D). Histologically, tumors were exclusively HB, with frequent Ki67 positive cells (Figure 3D).

**Table 1:** Detailed mouse data from YAP/β-catenin/pT3 EF1α (pT3) and YAP/β-catenin/pT3 EF1α Cre (Cre) injected *Raptor*^*fl/fl*^ mice

*Injection*	*Gender*	*weeks post injection*	*body weight (g)*	*liver weight (g)*	*macroscopic view*
YAP/β-catenin/pT3 EF1α	M	9	25.5	5.2	Tumor (whole liver)
	M	9.6	27	7.7	Tumor (whole liver)
	F	9.8	21.6	4.6	Tumor (whole liver)
	F	10.4	21.3	6.1	Tumor (whole liver)
	M	10.9	26.3	6.1	Tumor (whole liver)
	F	11	24	4.1	Tumor (whole liver)
	M	12.3	25.6	5.9	Tumor (whole liver)
YAP/β-catenin/pT3 EF1α Cre	F	10.1	25.2	1.1	Normal liver
	M	11	30	1.5	Normal liver
	M	11.3	26	1.6	Normal liver
	M	12.9	34.1	1.7	Normal liver
	M	15.3	34.1	1.7	A nodule (D=1.5mm)
	M	17.6	31.5	1.7	Nodule (N=4)
	F	20.3	25.4	1	Normal liver
	F	20.7	24.4	1	A nodule (D=1mm)
	M	21.3	31.4	1.6	Nodule (N=3)
	M	21.3	28.8	3	Nodule (N=8)

Abbreviations: D, diameter; N, number of nodules.

In summary, the present study demonstrates that ablation of *Raptor* strongly inhibits YAP/β-catenin driven HB formation in mice, supporting a major role of mTORC1 in HB.

### YAP and TAZ regulate mTOR activation *via* SLC38A1 along HB development

**Figure 4:**
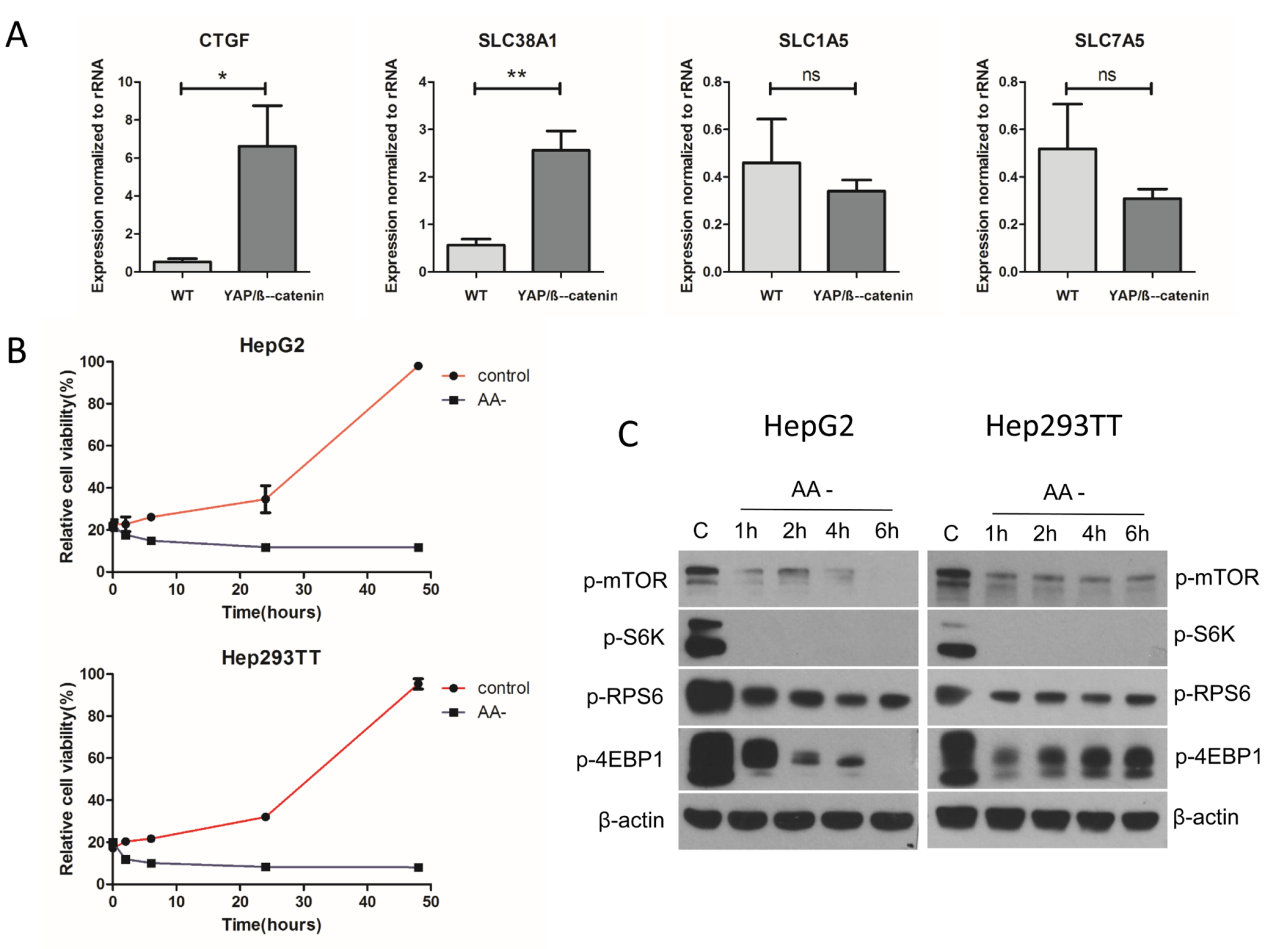
Amino acids are the major factor regulating mTORC1 function in the YAP/β-catenin-induced HB model **A.** Expression of CTGF, SLC38A1, SLC1A5, and SLC7A5 in wild type (WT) liver and YAP/β-catenin-induced HB by qRT-PCR. Data are presented as mean ± SEM. * *P* < 0.05, ** *P* < 0.01; ns, not significant. **B.** Relative cell viability in amino acid deficient (AA-) medium compared to that in regular medium (control). **C.** Western blotting showing that amino acid deprivation inhibits mTORC1 activation in HepG2 and Hep293TT cells.

Next, we investigated the possible mechanisms whereby mTORC1 is activated in HB. In this regard, a recent study suggested that YAP and TAZ regulate SLC38A1 and SLC7A5 expression in human HCC cells, leading to mTORC1 activation [22]. Therefore, we tested the hypothesis that YAP and TAZ may function to regulate mTORC1 in an analogous manner in HB. For this purpose, we first determined the expression of amino acid transporters SLC38A1, SLC7A5, and SLC1A5 in normal liver and YAP/β-catenin-induced HB tissues. Connective tissue growth factor (CTGF), a well-known target of YAP, was used as positive control (Figure 4A). Importantly, we found that in YAP/β-catenin-induced HB tumors, only SLC38A1 expression was upregulated (Figure 4A). Next, we investigated whether amino acids were required for human HB cell growth and mTORC1 activation. For this purpose, Hep293TT and HepG2 cells were cultured in amino acid free medium (Figure 4B and 4C). Of note, we found that deprivation of amino acids strongly inhibited Hep293TT and HepG2 cell growth (Figure 4B), and it led to the reduction of mTORC1 activation, as indicated by decreased p-mTOR, p-S6K, p-RPS6, and p-4EBP1 levels (Figure 4C). Thus, the results support the hypothesis that amino acids are key activators of mTORC1 in HB cells.

**Figure 5:**
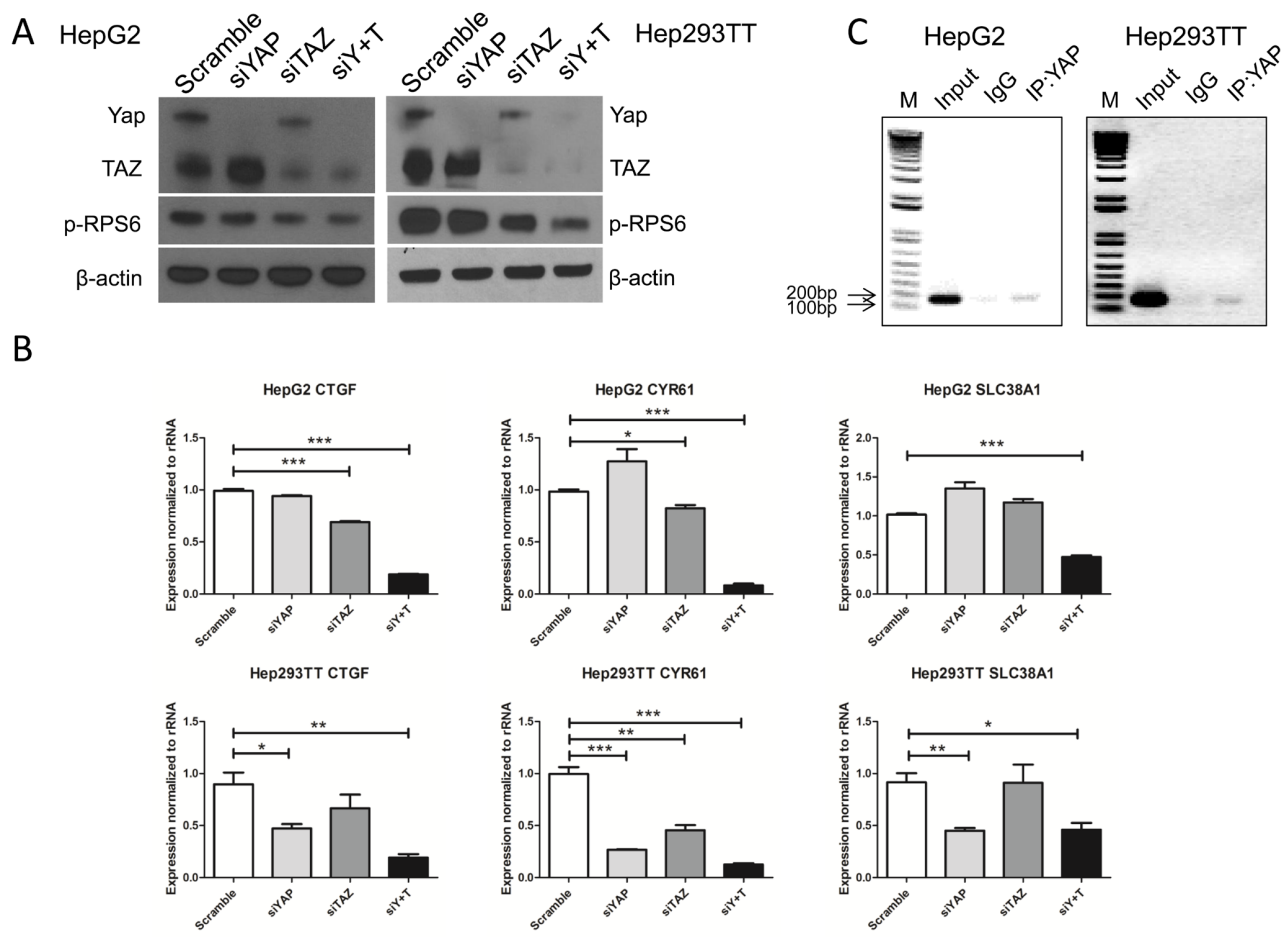
YAP and TAZ activate mTORC1 function *via* regulating SLC38A1 expression HepG2 and Hep293TT cells were transfected with indicated siRNA for 48-72 hours, siY+T: siYAP+siTAZ. **A.** Expression of YAP, TAZ and phosphorylated/activated (p)-RPS6 were measured by Western blotting. **B.** qRT-PCR was used to analyze CTGF, CYR61, and SLC38A1 expression. Data are presented as mean ± SEM. * *P* < 0.05, ** *P* < 0.01, *** *P* < 0.001. **C.** Direct binding of YAP to SLC38A1 promoter analyzed by chromatin immunoprecipitation (ChIP)-PCR in HepG2 and Hep293TT cells.

We further investigated whether YAP and TAZ control mTORC1 activity in HB cells. For this goal, we silenced YAP, either alone or in association with TAZ, using siRNAs in Hep293TT and HepG2 cells. We found that, indeed, when YAP and TAZ were concomitantly silenced, p-RPS6 levels were significantly decreased (Figure 5A). Next, we determined whether YAP and TAZ modulate SLC38A1 expression. Again, YAP alone, TAZ alone or YAP and TAZ together were silenced in Hep293TT and HepG2 cells. Expression of SLC38A1 and YAP/TAZ target genes (CTGF and cysteine-rich 61 or CYR61) was analyzed using qRT-PCR. As expected, concomitant silencing of YAP and TAZ triggered downregulation of CTGF, CYR61, and SLC38A1 in Hep293TT and HepG2 cells (Figure 5B). Finally, using chromatin immunoprecipitation (ChIP) assay, we found that YAP binds to the promoter region of SLC38A1 (Figure 5C) in HepG2 and Hep293TT cells, supporting the hypothesis that YAP directly regulates SLC38A1 expression in HB cells.

### β-catenin plays no significant role in regulating SLC38A1 levels in hepatoblatoma cells

**Figure 6:**
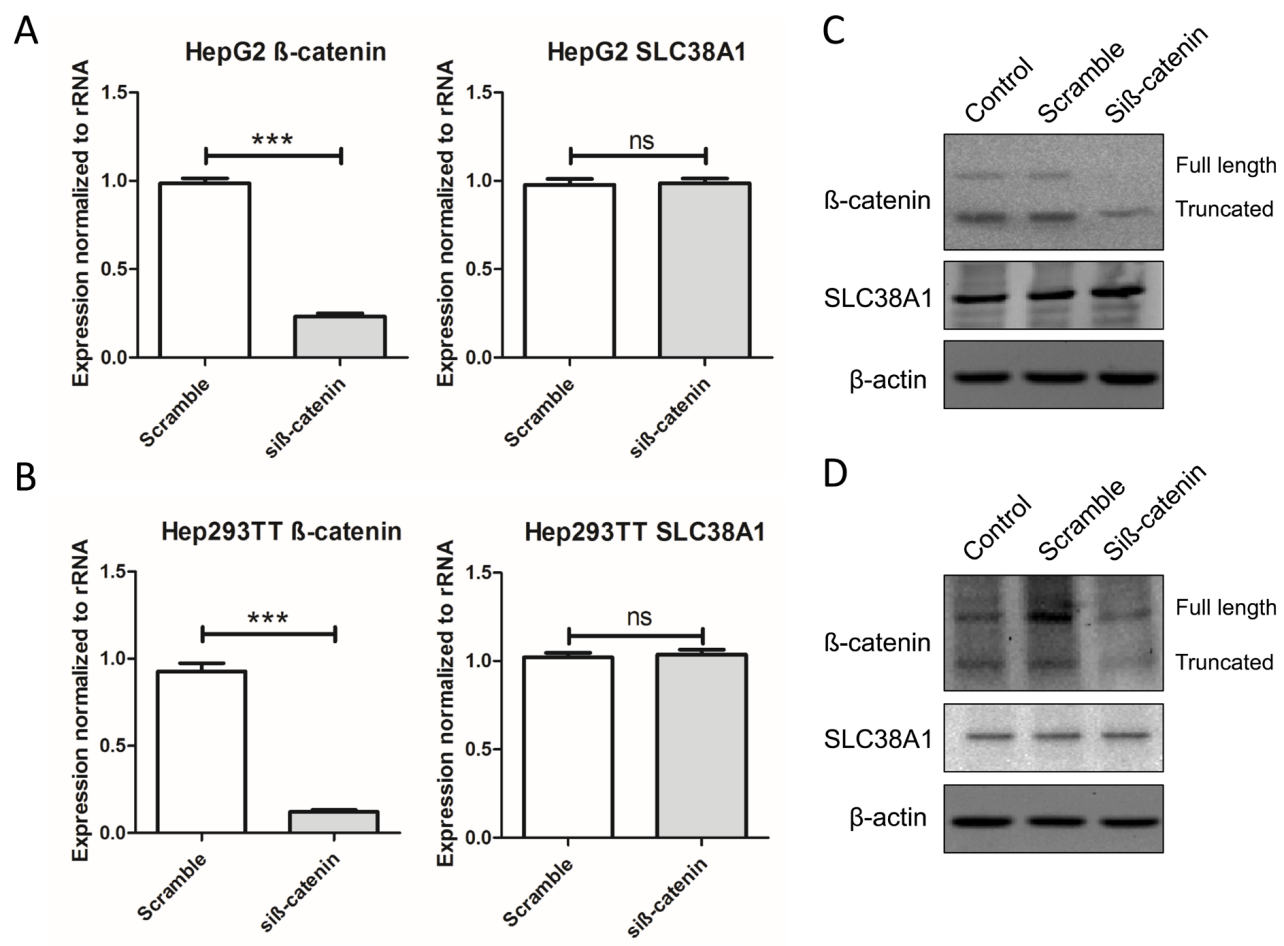
Genetic disruption of β-catenin does not affect SLC38A1 levels in HB cell lines SLC38A1 mRNA levels were detected following silencing of β-catenin in HepG2 **A.** and Hep293TT **B.** cell lines by qRT-PCR. Data are presented as mean ± SEM. *** *P* < 0.001, ns, not significant. No changes in SLC38A1 protein levels were detected in HepG2 **C.** and Hep293TT **D.** cell lines following silencing of β-catenin, as revealed by Western blotting.

Since YAP synergizes with β-catenin to induce HB formation, we next determined whether β-catenin also regulates SLC38A1 expression. However, no significant changes in SLC38A1 mRNA and protein levels were detected in HB cell lines upon β-catenin silencing (Figure 6), thus indicating that modulation of SLC38A1 expression is β-catenin-independent in HB cells.

### mTORC1 signaling and SLC38A1 are concomitantly activated in human hepatoblastoma

**Figure 7:**
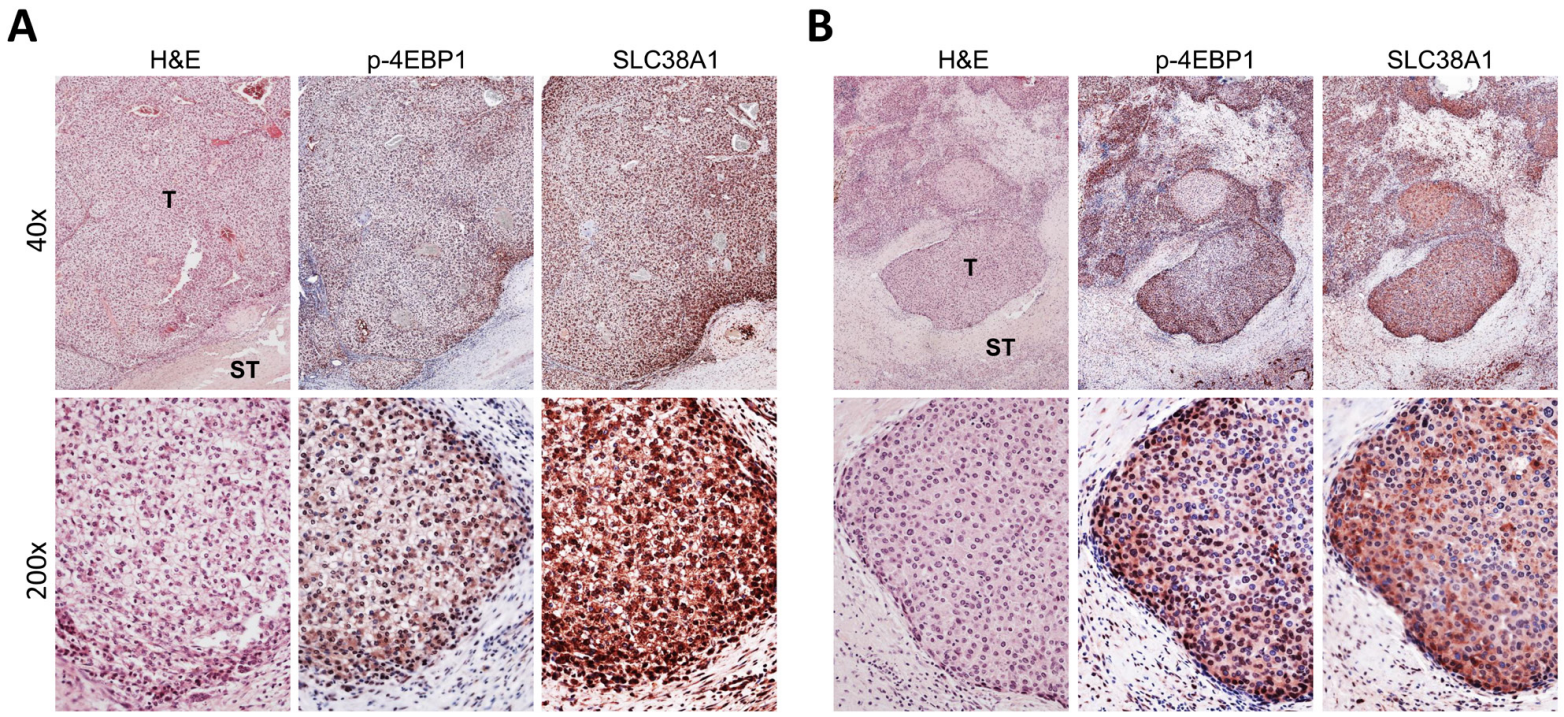
Frequent concomitant activation of mTORC1 and SLC38A1 in human hepatoblastoma Immunohistochemical pattern of phosphorylated/inactivated (p-)4EBP1, a surrogate marker of mTORC1 activation, and SLC38A1 in two human hepatoblatoma specimens. The two hepatoblastomas **A.**, **B.** are depicted in two magnifications (40X and 200X; upper and lower panels, respectively) and show strong immunoreactivity for both mTORC1 and SLC38A1 in the tumor part (T) when compared with non-tumorous surrounding liver tissues (ST). H&E, haematoxylin and eosin staining.

Finally, we assessed the levels of p-4EBP1, a surrogate marker of mTORC1 activation, and SLC38A1 in a collection of human HB specimens (*n* = 28) by immunohistochemistry (Figure 7). Strikingly, strong immunoreactivity for p-4EBP1 and SLC38A1 proteins was detected in most tumor tissues when compared with corresponding non-tumorous surrounding liver tissues (Figure 7). Specifically, increased levels of SLC38A1 and p-4EBP1 were detected in 20 of 28 (71.4%) and 23 of 28 (82.1%) HB specimens, respectively. Of note, 18 of 20 (90%) HB showing upregulation of SLC38A1 also displayed elevated levels of p-4EBP1. No significant association was found between the levels of SLC38A1 and/or p-4EBP1 with clinicopathologic features of the patients, including age, gender, etiology, histology subtype, recurrence or lung metastasis (data not shown).

**Figure 8:**
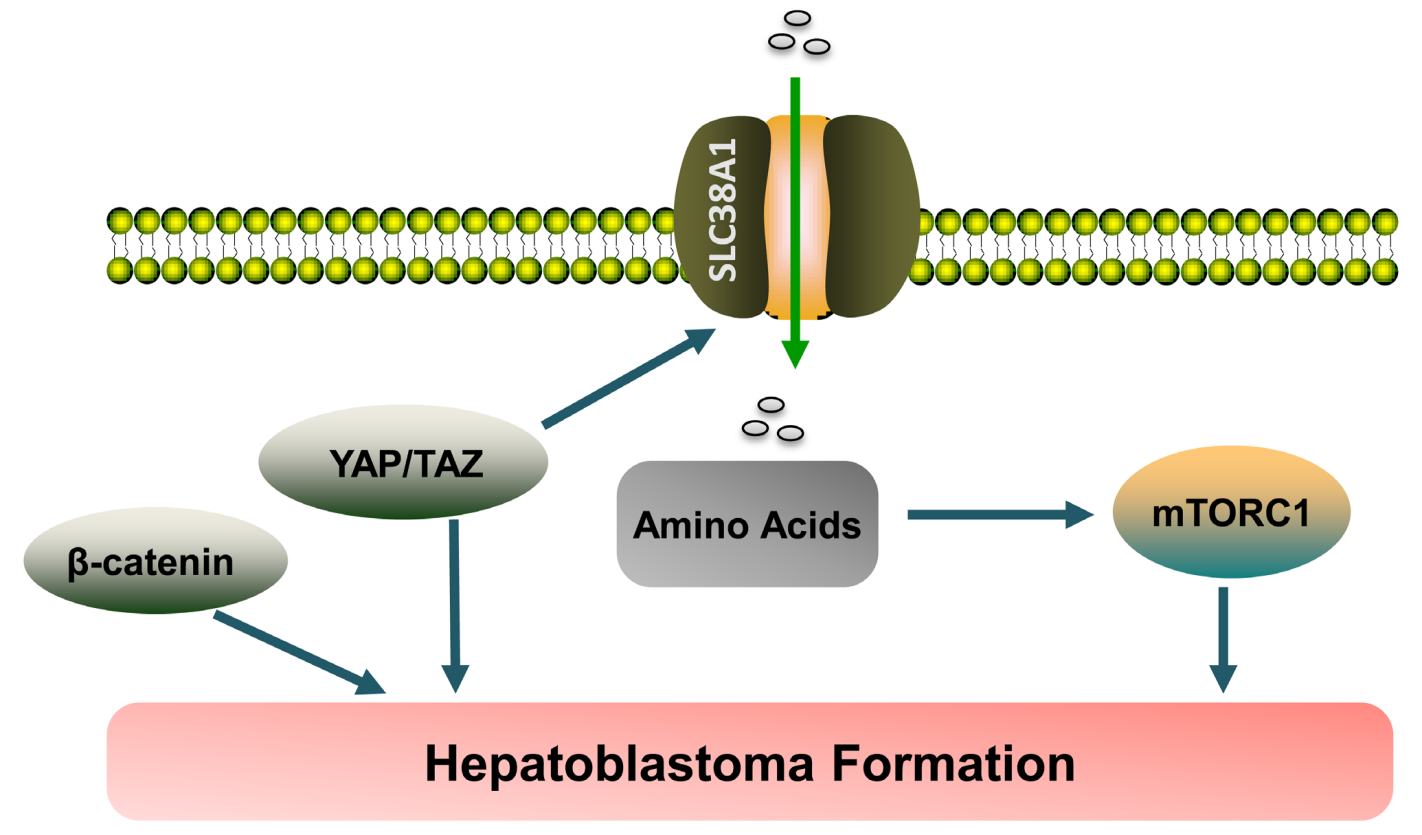
Pathway illustration YAP and its paralog TAZ regulate the expression of the amino acid transporter SLC38A1, thus leading to mTORC1 activation and hepatoblastoma formation.

In summary, our comprehensive studies demonstrate that YAP and TAZ positively modulate mTORC1 activity *via* SLC38A1 mediated amino acid uptake and promote HB pathogenesis both in human and in mice (Figure 8).

## DISCUSSION

HB is a malignant form of liver cancer occurring in infants and children [1, 2]. Similar to other tumor types, multiple genetic alternations are likely to be required for the development of HB. In particular, somatic mutations of the *CNNTB1* gene leading to the activation of Wnt/β-catenin signaling cascade, are a well-documented major driver genetic event in HB development [4, 5]. A recent body of evidence indicates that YAP is another, pivotal factor commonly activated in HB [11]. In particular, it has been shown that concomitant activation of β-catenin and YAP results in HB formation in mice [11]. Also, silencing of YAP and/or β-catenin leads to decreased proliferation of HB cell lines [11]. However, the precise mechanisms by which YAP promotes HB development are not known. In this study, we demonstrate that mTORC1 is activated in both human HB cell lines and mouse YAP/β-catenin-induced tumor tissues. The importance of the mTORC1 pathway along HB development is underscored by the finding that inhibition of mTORC1 by either MLN0128 treatment or ablation of *Raptor* strongly suppresses HB occurrence in mice. Furthermore, we showed that YAP and its paralog TAZ regulate the expression of the amino acid transporter SLC38A1 in human HB cell lines, leading to mTORC1 activation (Figure 8). Our investigation therefore provides novel mechanistic insight into how YAP and TAZ may contribute to HB formation.

The mTOR pathway is a major signaling cascade regulating tumor cell growth and metabolism [12]. Thus, targeting the mTOR pathway is considered to be a valid therapeutic approach for cancer treatment [17]. Studies of mTOR pathway in HB are still scanty. Hartmann *et al.* reported that in 24 human HB specimens, p-mTOR expression was detected in 96% (23/24) cases [23]. Similarly, in the present study, we show the activation of mTORC1 (as revealed by p-4EBP1 immunohistochemistry) in the large majority of HB specimens from an independent collection. In addition, it has been found that Rapamycin inhibits proliferation and induces apoptosis in HepG2, HepT1, and Huh6 human HB cells *in vitro*. Rapamycin also suppresses Huh6 growth in a xenograft model [24]. Altogether, these data indicate that mTOR, and particularly mTORC1, is frequently activated in human HB. Thus, targeting mTORC1 might be beneficial for HB treatment. However, the mechanisms promoting mTORC1 activation in HB were not investigated to date. In our current investigation, we provide a novel mechanism by which YAP and TAZ activate mTORC1, namely *via* transcriptional upregulation of SLC38A1. In this regard, the concordant data obtained in the YAP/β-catenin mouse model, HB cell lines, and human HB specimens strongly suggest that the same mechanism of mTORC1 induction is at play along murine and human HB development. Nonetheless, larger human HB collections should be analyzed to further substantiate the present findings. Although we found that YAP contributes to HB development *via* activation of mTORC1, we cannot exclude that YAP promotes HB occurrence also through additional molecular mechanisms. For instance, it has been recently shown that the transcription factor Forkhead Box M1 (FOXM1) is a major player along hepatocellular carcinoma development driven by YAP overexpression [25]. Preliminary data from our laboratory show that the functional interplay between YAP and FOXM1 is also present in human HB cell lines (Supplementary Figure 1). Additional investigation is required to determine the importance of FOXM1 in YAP-dependent HB development.

Furthermore, our data suggest the functional redundancy of YAP and its paralog TAZ in regulating SLC38A1 expression and mTORC1 activity in human HB cells. While the relevance of YAP in HB is underscored by a body of experimental evidence, including the findings from the present study, there is no study on the role of TAZ in this tumor type. Our results support the possible involvement of TAZ in HB, and this issue clearly warrants further studies. In particular, it would be highly important to determine whether YAP and TAZ, besides SLC38A1 and mTORC1, regulate the same or distinct pathways in HB.

Solute carrier (SLC) proteins are large families of membrane transporters involving multiple cellular activities, such as nutrient uptake, waste removal, and iron transport [26]. Recent studies have indicated the key roles of amino acid transporters along tumor development [27], especially in regulating the mTOR pathway [28]. For instance, a recent work from our group indicates that SLC1A5 and SLC7A6 are direct targets of c-Myc and are required for c-Myc induced mTORC1 activation in HCC [29]. Also, SLC1A5 mediated glutamine uptake was found to be required for lung cancer cell growth [30]. The functional roles of amino acid transporters in HB formation are not well characterized. In this study, we demonstrate that SLC38A1 is regulated by YAP and TAZ in HB cells. Unlike YAP and TAZ, which are nuclear proteins and are difficult to be targeted by small molecules or antibody antagonists, SLC families of transporter proteins are clearly druggable. Indeed, SLC targeting drugs have been widely used for the treatment of multiple diseases, including, central nervous system disorders, cardiovascular disease, and antineoplastic treatment [31]. Thus, our study supports the development of drugs that target SLC38A1 for HB therapy.

## MATERIALS AND METHODS

### Construct and reagents

The construct used for the mice, including pT3-EF1α-YAPS127A, pT3-EF1α-ΔN90-β-catenin, pT3-EF1α, pT3-EF1α-Cre, and pCMV/sleeping beauty transposase (SB), were described previously [11, 21]. All the plasmids were purified using the Endotoxin free Maxi prep kit (Sigma-Aldrich, St. Louis, MO, USA).

### Mice and hydrodynamic transfection

Wild-type FVB/N mice were obtained from Jackson Laboratory (Bar Harbor, ME). *Raptor*^*fl/+*^ mice [32] were purchased from the Jackson Laboratory (Bar Harbor, ME, USA; stock: 013188) and intercrossed to generate *Raptor*^*fl/fl*^ mice. SB-mediated hydrodynamic injections were performed as described [33]. Briefly, for the HB tumorigenesis model, 20 μg of pT3-EF1α-YAPS127A and 20 μg of pT3-EF1αH-ΔN90-β-catenin along with SB transposase in a ratio of 25:1 were delivered into the FVB/N mouse by hydrodynamic injection. These two plasmids contain constitutively active forms of YAP and β-catenin protooncogenes. For further knocking out *Raptor* in the HB model, either 40μg pT3-EF1a or 40μg pT3-EF1a-Cre were co-injected with 20μg pT3-EF1α-YAPS127A and 20μg pT3-EF1α-ΔN90-beta-catenin together as well as 3.2μg pCMV-SB in *Raptor*^*fl/fl*^ mice. All mice were monitored closely and euthanized as described in the main text. Mice were fed and monitored according to protocols approved by the Committee for Animal Research at the University of California, San Francisco.

### Human HB cell lines and *in vitro* treatments

The Hep293TT cell line [18] was kindly provided to us by Dr. Gail Tomlinson from the University of Texas Southwestern Medical Center. Hep293TT cells were cultured in Roswell Park Memorial Institute (RPMI) 1640 medium, 25mM Hepes, 10% fetal bovine serum (FBS) and 1% penicillin/ streptomycin (Gibco). HepG2 and SNU449 cells were purchased from ATCC and they were cultured in Dulbecco’s modified Eagle medium (DMEM) supplemented with 10% FBS and 1% penicillin/ streptomycin. To block mTORC1 signaling, MLN0128 (LC Laboratories), a second generation of mTOR kinase inhibitor was diluted to gradient concentration and added to the cells. Dimethyl sulfoxide (DMSO) was diluted and used as control. After 48 hours treatment, cells were stained with crystal violet or collected for protein analysis. Cell proliferation and apoptosis were assessed using the BrdU Cell Proliferation Assay Kit (Cell Signaling Technology Inc) and the Cell Death Detection Elisa Plus Kit (Roche Molecular Biochemicals, Indianapolis, IN), respectively, following the manufacturers’ instructions.

For amino acid deprivation experiments, Amino Acid Free Medium (US Biological, Salem, MA) was added to the cells. Subsequently, cells were stained with crystal violet or collected for protein analysis. For gene silencing studies, cells were plated in 6-well plates and transfected with 30 pmol siRNA targeting YAP (ID# s20366; ThermoFisher Scientific, Waltham, MA), TAZ (ID# s24789; ThermoFisher Scientific), TEAD4 (ID# 107036; ThermoFisher Scientific), and β-catenin (ID# AM51331; ThermoFisher Scientific), either alone or in combination using Lipofectamine RNAiMAX (Life Technologies) according to the manufacturer’s instructions. A scramble small interfering RNA (siRNA; ID# 4390844; ThermoFisher Scientific) was used as negative control RNA. 48-72h post transfection, cells were collected for protein and RNA analysis. Experiments were repeated at least three times in triplicate.

### Chromatin immunoprecipitation assay

Cells were crosslinked in 1% formaldehyde (Polysciences, Inc. #18814) for 7 min at room temperature. After glycine quenching, cell pellets were collected, and sonicated using the Diagenode Bioruptor. Sonication was performed at high power, 30s on, 30s off, 5 min per cycle, for a total of 3 cycles. The sheared chromatin was incubated with protein A beads (ZYMO Research, Irvine, CA) and antibodies (YAP, 14074, Cell Signaling Technology; or Rabbit IgG, 2729, Cell Signaling Technology). DNA was purified using the Zymo-Spin ChIP Kit (D5210) and quantified by PCR. The primer sequences used for PCR were: human SLC38A1 promoter region primer forward (F): 5’- CAAGATTTGGATGTGCCACTTAG -3’; human SLC38A1 promoter region primer reverse (R): 5’- TGATTCCTCTATTCACTGTGTGCT-3’.

### Protein extraction and Western blotting

Liver tissues or cell pellets were homogenized or suspended in lysis buffer [30 mM Tris (pH 7.5), 150 mM NaCl, 1% NP-40, 0.5% Na deoxycholate, 0.1% SDS, 10% glycerol, and 2mM EDTA] containing the Complete Protease Inhibitor Cocktail (ThermoFisher Scientific). Protein concentrations were determined with the Bio-Rad Protein Assay Kit (Bio-Rad, Hercules, CA) using bovine serum albumin as a standard. Aliquots of 30 μg lysates were denatured by boiling in Tris-Glycine SDS Sample Buffer (Bio-Rad), separated by SDS-PAGE, and transferred to nitrocellulose membranes (Bio-Rad) by electroblotting. Membranes were blocked in 5% non-fat dry milk in Tris-buffered saline containing 0.1% Tween 20 for 1 hour and probed with following specific antibodies: AKT (9272), Phospho-AKT(S473) (4060), Phospho-AKT(T308) (13038), Phospho-mTOR (2971), Phospho-p70 S6 Kinase (9205), Phospho-RPS6 (4858), Phospho-4E-BP1(T37/46) (2855), Phospho-4E-BP1(S65) (9451), Phospho-NDRG1 (5482), Survivin (2808), YAP/TAZ (8418), Cleaved Caspase-3 (CC-3) (9664), Mcl-1 (94296), Bcl-Xl (2764), Bak (12105; Cell Signaling Technology), SLC38A1 (HPA052272; Sigma-Aldrich, St. Louis, MO). Anti-β-catenin antibody (610153) was purchased from BD Biosciences (Franklin Lakes, NJ). Anti-GAPDH (MAB374; EMD Millipore, Billerica, MA), anti-β-Actin (A5441; Sigma-Aldrich, St. Louis, MO), and anti-β-tubulin (6046; Abcam) antibodies were used as loading controls. Each primary antibody was followed by incubation with horseradish peroxidase-secondary antibody (Jackson ImmunoResearch Laboratories Inc., West Grove, PA) diluted 1:10000 for 1 hour and then visualized by the Super Signal West Dura (ThermoFisher Scientific). For nuclear protein extraction, proteins were extracted by using the Cytoplasmic Extraction Reagent I and II (ThermoFisher Scientific). After thoroughly removing the supernatant (cytoplasmic extract), Nuclear Extraction Reagent (ThermoFisher Scientific) was added for the nuclear protein extraction. Aliquots of 5-10 μg of nuclear and cytoplasmic lysates were used for Western blotting. Isolation of cytoplasmic proteins was validated by β-tubulin, while Histone H3 (D1H2) (4499; Cell Signaling Technology) was used as loading control of nuclear proteins.

### Quantitative reverse transcription real-time polymerase chain reaction (qRT-PCR)

qRT-PCR reactions were performed with 100 ng of cDNA on the whole sample collection and cell lines, using an ABI Prism 7000 Sequence Detection System and TaqMan Universal PCR Master Mix (ThermoFisher Scientific). Reaction conditions were: 10 min of denaturation at 95°C and 40 cycles at 95°C for 15 s and at 60°C for 1 min. Quantitative values were calculated by the PE Biosystems Analysis software and expressed as N target (NT). NT = 2^-ΔCt^, wherein ΔCt value of each sample was calculated by subtracting the average Ct value of the target gene from the average Ct value of the ribosomal RNA (rRNA) gene. Primers used in the study include: 18S rRNA Forward (F): 5’-CGGCTACCACATCCAAGGAA-3’, Reverse (R): 5’-GCTGGAATTACCGCGGCT-3’; Mouse CTGF: F: 5’-GGGCCTCTTCTGCGATTTC-3’, R: 5’- ATCCAGGCAAGTGCATTGGTA-3’; Mouse SLC38A1: F: 5′-AGCAACGACTCTAATGACTTCAC-3’, R: 5’-CCTCCTACTCTCCCGATCTGA-3’; Mouse SLC7A5: F: 5′-CTACGCCTACATGCTGGAGG-3’, R: 5’-GAGGGCCGAATGATGAGCAG-3’; Mouse SLC1A5: F: 5′-TTCGCTATCGTCTTTGGTGTG-3’, R: 5’-ATGGTGGCATCATTGAAGGAG-3’; Human CTGF: F: 5′-CAGCATGGACGTTCGTCTG-3’, R: 5’-AACCACGGTTTGGTCCTTGG-3’; Human CYR61: F: 5′-GGTCAAAGTTACCGGGCAGT-3’, R: 5’-GGAGGCATCGAATCCCAGC-3’; Human SLC38A1: F: 5′-AACCTCCTTAGGCATGTCTGT-3’, R: 5’-GCAAAGGCGAGTCCCAAAAT-3’; Human YAP1: F: 5’-TAGCCCTGCGTAGCCAGTTA-3’, R: 5’-TCATGCTTAGTCCACTGTCTGT-3’; Human TEAD4: F: 5’-GGACACTACTCTTACCGCATCC-3’, R: 5’-TCAAAGACATAGGCAATGCACA-3’; Human FOXM1: F: 5’-TTGCCCGAGCACTTGGAATC-3’, R: 5’-GTATGAGCTGACCCGTGGT-3’. Predesigned primers for human β-catenin (ID# Hs00355049_m1) were purchased from Applied Biosystems (Foster City, CA).

### Immunohistochemistry

Liver specimens were fixed in 4% paraformaldehyde overnight at 4°C and embedded in paraffin. Hematoxylin & Eosin (H&E) staining on 4μm liver sections was performed to characterize histopathologically liver preneoplastic and neoplastic lesions in mice. Immunohistochemistry was performed as described previously [29]. Briefly, antigen retrieval was achieved in deparaffinized sections by boiling in 10mM sodium citrate buffer (pH 6.0) for 10 min. After a blocking step with the 5% goat serum and Avidin-Biotin blocking kit (Vector Laboratories, Burlingame, CA), the slides were incubated with primary antibodies overnight at 4°C. Primary antibodies used for the experiment are as follows: p-RPS6 (4858; Cell Signaling Technology), Ki67 (RM-9106; Thermo Fisher Scientific), p-4EBP1 (2855; Cell Signaling Technology), and SLC38A1 (HPA052272; Sigma-Aldrich, St. Louis, MO). These primary antibodies were selected for the analysis since they have been extensively validated by the manufacturers for immunohistochemistry. After washes, slides were incubated in 3% H2O2 for 20 minutes to quench the endogenous peroxidase, then followed by one hour of secondary antibody incubation. Signal was detected by the Vectastain ABC Elite Kit (Vector Laboratories) and visualized by DAB (Vector Laboratories).

### Human tissue samples

A collection of formalin-fixed, paraffin-embedded human HB (*n* = 28) samples was used in the present study. The clinicopathological features of liver cancer patients are summarized in Table 2. HB specimens were collected at the Medical University of Greifswald (Greifswald, Germany) and from the Archives of the Pathology Departments of Semmelweis University (Budapest, Hungary). Institutional Review Board approval was obtained at the local Ethical Committee of the Medical University of Greifswald and the Regional Ethical Committee of the Semmelweis University. Informed consent was obtained from all subjects.

**Table 2:** Clinicopathological features of hepatoblastoma (HB) patients

Variables	
No. of patients Male Female	28 16 12
Age (years) Mean ± SD	4.4 ± 2.8
Tumor morphology Fetal Embrional Mixed	14 8 6
Recurrence Yes No	10 18
Lung metastases Yes No	9 19

### Statistical analysis

All data are presented as mean ± SEM. Statistical analysis was performed using two-tailed unpaired t test or Tukey-Kramer test. *P* value < 0.05 was considered significant. Overall survival was estimated according to Kaplan-Meier and Log-rank (Mantel-Cox) test. All statistics were performed with Prism 6, version 6.0 (GraphPad Software Inc., La Jolla, CA).

## SUPPLEMENTARY MATERIALS FIGURE



## Abbreviations

CTGF: connective tissue growth factor
CYR61: cysteine-rich 61
HB: Hepatoblastoma
HCC: Hepatocellular carcinoma
mTOR: mammalian target of rapamycin
mTORC1: mammalian target of rapamycin complex 1
mTORC2: mammalian target of rapamycin complex 2
SLC: Solute carrier
TAZ: transcriptional co-activator with PDZ binding motif
YAP: Yes-associated protein
