# Supplementary material for: Central role of mTORC1 downstream of YAP/TAZ in hepatoblastoma development
Source: Oncotarget. 2017 Sep 1;8(43):73433–47. doi: 10.18632/oncotarget.20622 (PMC5650273; doi:10.18632/oncotarget.20622)
Supplement: Supplementary file 1 [file oncotarget-08-73433-s001.pdf]

Central role of mTORC1 downstream of YAP/TAZ in hepatoblastoma development

SUPPLEMENTARY MATERIALS

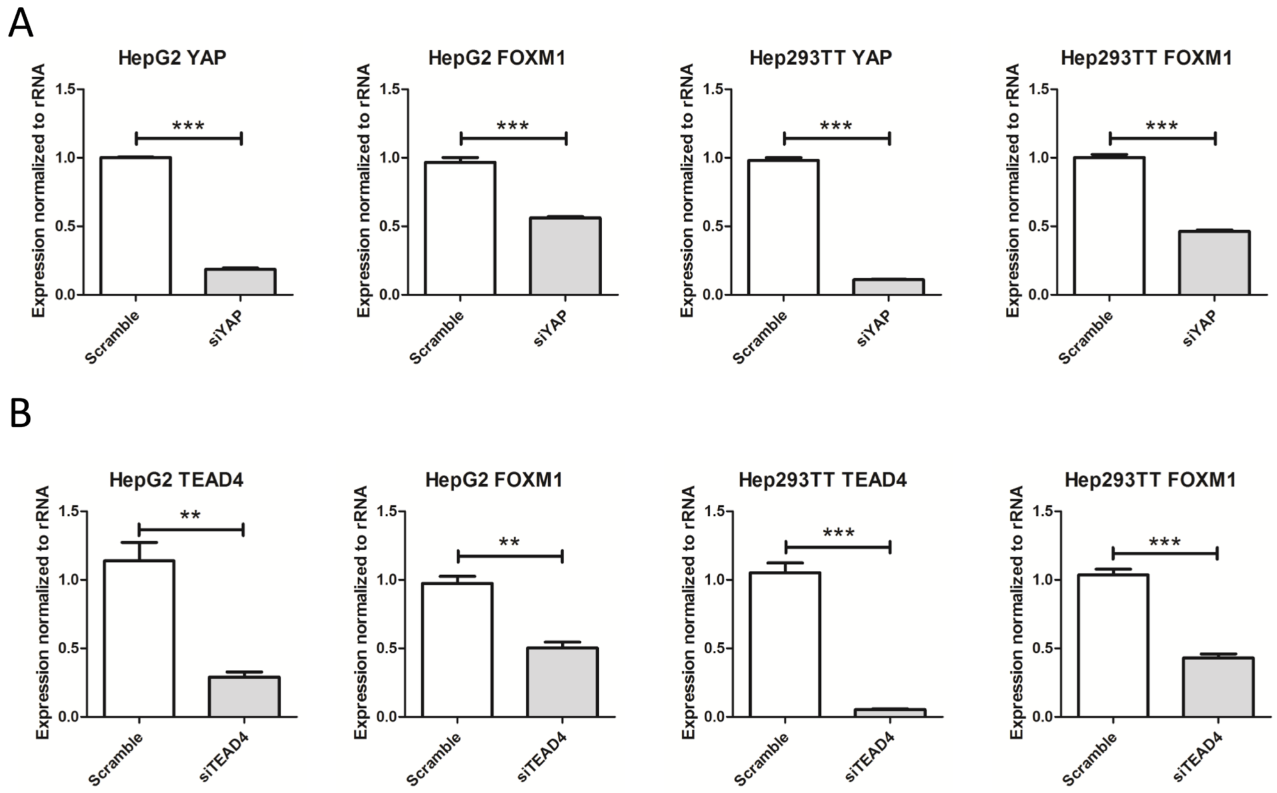

Supplementary Figure 1: Silencing YAP or TEAD4 downregulates the expression of FOXM1 in HB cells. qRT- PCR analysis showing decreased FOXM1 expression following silencing YAP (**A**) or TEAD4 (**B**) in HepG2 and Hep293TT cell lines. Data are presented as mean + SEM. " P<0.01, "*" P<0.001
